# Mechanisms creating homogamy in depressiveness in couples: A longitudinal study from Czechia

**DOI:** 10.1038/s41598-025-93065-7

**Published:** 2025-03-17

**Authors:** Zsófia Csajbók, Jakub Fořt, Peter K. Jonason, Jan Havlíček, Jakub Binter, Zuzana Štěrbová

**Affiliations:** 1 Department of Psychology and Life Sciences, Faculty of Humanities, Charles University, Pátkova 5, 182 00 Prague 8, Czech Republic; 2 Department of Zoology, Faculty of Science, Charles University, Prague, Czech Republic; 3 Psychology Research Institute, University of Economics and Human Sciences, Warsaw, Poland; 4 Department of Regional Development and Public Administration, Faculty of Social and Economic Studies, University of Jan Evangelista Purkyně, Ústí nad Labem, Czech Republic; 5 Department of Psychology, Faculty of Arts, Charles University, Prague, Czech Republic; 6 Department of Philosophy and History of Science, Faculty of Science, Charles University, Prague, Czech Republic

**Keywords:** Depressiveness, Homogamy, Heterogamy, Assortative mating, Mate preference, Convergence, Sexual selection, Human behaviour, Translational research

## Abstract

Couples often resemble each other in characteristics like depression, but the reasons for this homogamy (i.e., similarity) remain unclear. We investigated two potential mechanisms: preference for a self-similar partner and convergence (i.e., increasing similarity) over time. In a nationally representative sample of 2,793 Czech individuals who we surveyed three times in one year, we examined self-reports of participants’, their ideal partners’, and their actual partners’ “pessimism and depressiveness”. Participants preferred partners less depressive than themselves, yet their actual partners were more depressive than desired. Those who ended their relationships showed a greater ideal-versus-actual partner discrepancy than those who stayed together. In stable relationships, individuals adjusted their ideal preferences to align more closely with their actual partners over time. We identified four relationship classes with latent class growth modeling based on self and partner evaluations: both non-depressive, both depressive, self depressive and partner non-depressive, and self non-depressive and partner depressive. Romantic relationships were most stable when both partners were non-depressive and most likely to dissolve when both were depressive. While we failed to detect convergence overall, we found it within heterogamous (i.e., dissimilar) classes. Overall, our findings suggest that homogamy and heterogamy in depressiveness are complexly associated with relationship maintenance.

**Supplementary Information:**

The online version contains supplementary material available at 10.1038/s41598-025-93065-7.

## Introduction

Depression is a pervasive and common mental health issue^1^. One potentially influential and not well-understood factor in the etiology of depression is the presence of depression in one’s romantic partner. Several undesirable effects of one or both partners’ depression in a romantic relationship have been identified, including emotional toll, lack of energy, impaired communication, sexual life, and relationship satisfaction^2,3^. Further, offspring in families where both parents suffer from depression have an increased risk of developing depression themselves and experiencing an earlier onset^4^. Therefore, understanding how these relationships are formed could inform us about possible supportive measures.

Romantic partners tend to resemble each other in many psychological traits and mental disorders, such as affective disorders including depression^5–9^, and personality traits including pessimism^10^. Although partner similarity in depression is well-established, the mechanisms underlying this homogamy (i.e., the tendency to couple with a self-similar partner) remain generally unclear, despite several plausible explanations. Four mechanisms can lead to homogamy^11,12^: (1) *mating market operations* (i.e., to accept a partner that is similar for the lack of mutual attraction from more desirable partners); (2) *proximity* or social homogamy (i.e., potential mates meet each other from similar backgrounds); (3) *preference* and active choice for self-similarity; and (4) *convergence* over time (i.e., coupled individuals share the same routines and influence each other in their attitudes, behavior, etc.).

To the best of our knowledge, only one published study has examined preferences for personality traits partially associated with depressiveness that are negatively valenced and potentially maladaptive^13^. The study found medium positive associations (*r*s = 0.17 to 0.37) between self-rating and partner preference for neuroticism, detachment, closedness, antagonism, disinhibition, and psychoticism but no relationship for negative affectivity, which is related to depression and persistent depressive disorder^14,15^. Additionally, no convergence was found in depression by some^16,17^, but others found that women’s elevated depression subsequently increased men’s depression over time^18–20^, or that men’s depression increased women’s depression^21^, or that they equally influenced each other’s depression^22^. Thus, the limited research so far indicates that individuals prefer similar partners in negatively valenced personality traits which are unrelated to depressiveness. However, no such evidence was found in negative affectivity, a characteristic directly related to depression and depressiveness. Also, results are mixed concerning whether similarity is a consequence of convergence over time.

### Overview

Although homogamy in depression is well-documented, few have examined the ostensible underlying mechanisms. For instance, mate choice (as opposed to preferences) in depression-related characteristics have yet to be observed in longitudinal data. Thus, to investigate two of the possible etiological mechanisms for romantic couples’ similarity in depressiveness, we focused on preference for self-similar partners and convergence over time. This study was part of a larger project called “Dynamics of Czech Romantic Relationships” where other variables were measured on partner preferences and romantic relationship experiences^23^. Thus, we operationalized depressiveness with a single item (for the sake of brevity) rating “pessimism and depressiveness” instead of studying “depression” per se. We did so, because we assumed that depressiveness (i.e., depressive traits) are relatively stable in comparison to depression, which can improve or worsen for many reasons outside the relationship context^15,24,25^. In other words, while participants may perceive depression as a disorder requiring treatment, depressiveness could be seen as a more persistent and inherent characteristic of a potential partner in a subclinical way. On the other hand, we relied on literature and evidence obtained on depression, as trait-like depressiveness concepts have not been studied widely in the context of romantic relationships (but see^13^). We tested differences between the ratings of “pessimism and depressiveness” in an ideal partner, one’s actual partner, and participants’ themselves across three waves that were approximately half a year apart overall and in men and women separately. We assumed that on average women are more demanding in their partner preferences than men are^26–28^, thus they would prefer a less pessimistic and depressive partner than men.

Further, we focused on individuals who continued their relationships, experienced the dissolution of their relationships, or transitioned from singlehood to partnerships over our study period. This way, we captured how depressive traits are considered in mate choice processes that have not been investigated before. Lastly, we extracted distinct longitudinal trajectories of depressive traits. We expected that different trajectories, if found, could be associated with different relationship maintenance (e.g., staying together or breaking up) because of homogamy or heterogamy (i.e., the tendency to couple with a self-dissimilar partner) in depressiveness.

## Results

First, we compared the self, ideal partner, and actual partner ratings in depressiveness in each wave using SPSS (version 27) with Pearson correlations and paired samples *t*-tests instead of using ANOVAs to maximize the number of participants included (Table 1). Correlations between self-ratings and ideal partner ratings ranged between 0.32 and 0.39. Correlations between self-ratings and actual partner ratings ranged between 0.26 and 0.55. Correlations between actual and ideal partner ratings ranged between 0.50 and 0.59. There was no difference in correlations across heterosexual and non-heterosexual participants (Table S2). Participants rated their ideal partners as less depressive than themselves (Cohen’s *d*s = -0.43 to -0.52). They also rated their actual partners as less depressive than themselves except actual partner 2 in wave 3 (*d*s = -0.01 to 0.25). The ideal partners were less depressive than the actual partners (*d*s = -0.20 to -0.36).

**Table 1:** Self, ideal partner, and actual partner ratings of depressiveness and their comparisons.

			Mean	SD	*n*	*r*	t	Cohen’s d
Ideal partner v. Self	Ideal partner	Wave 1	1.88	1.33	2559	0.39***	– 21.70***	– 0.43
	Self-rating		2.58	1.60				
	Ideal partner	Wave 2	1.99	1.31	1844	0.32***	– 22.28***	– 0.52
	Self-rating		2.89	1.63				
	Ideal partner	Wave 3	1.99	1.28	1504	0.37***	– 18.88***	– 0.49
	Self-rating		2.77	1.56				
Self v. Actual partner	Self-rating	Wave 1	2.47	1.55	1959	0.30***	3.67***	0.08
	Actual partner 1		2.32	1.53				
	Self-rating	Wave 2	2.77	1.56	1205	0.26***	8.74***	0.25
	Actual partner 1		2.31	1.47				
	Self-rating	Wave 3	2.64	1.53	897	0.33***	6.00***	0.20
	Actual partner 1		2.29	1.49				
	Self-rating	Wave 2	2.81	1.62	285	0.33***	3.18**	0.19
	Actual partner 2		2.45	1.70				
	Self-rating	Wave 3	2.68	1.54	152	0.55***	– 0.15	– 0.01
	Actual partner 2		2.70	1.78				
Ideal partner v. Actual partner	Ideal partner	Wave 1	1.81	1.29	1959	0.50***	– 15.78***	– 0.36
	Actual partner 1		2.32	1.53				
	Ideal partner	Wave 2	1.98	1.30	1202	0.51***	– 8.19***	– 0.24
	Actual partner 1		2.30	1.47				
	Ideal partner	Wave 3	1.95	1.26	896	0.52***	– 7.66***	– 0.26
	Actual partner 1		2.29	1.49				
	Ideal partner	Wave 2	2.14	1.54	285	0.54***	– 3.39***	– 0.20
	Actual partner 2		2.45	1.70				
	Ideal partner	Wave 3	2.18	1.49	152	0.59***	– 4.22***	– 0.34
	Actual partner 2		2.70	1.78				

Partner 1 was tracked from wave 1, Partner 2 was tracked from wave 2.

**p < 0.01, ***p < 0.001

Second, we compared preferences for depressiveness and self- and actual partner ratings in depressiveness between men and women (Table 2). We found that women wanted less depressive partners than men in all three waves (*d*s =** -**0.20 to − 0.25). In contrast, women evaluated themselves slightly less depressive than men did in waves 1 and 2 (*d*s = − 0.10). Conversely, men evaluated their (predominantly female) actual partner 1 as more depressive than women in all three waves (*d*s = − 0.15 to − 0.26). That is, for example in wave 1, while women’s mean self-rating was 2.50 (*SD* = 1.58), men also rated their actual partner 2.50 (*SD* = 1.57) on average in depressiveness. In contrast, men’s mean self-rating was 2.66 (*SD* = 1.62), while women’s mean (predominantly male) partner rating was 2.18 (*SD* = 1.49; *d* = 0.31). Across men and women, we also compared the correlations with Fisher’s *z* tests and discrepancies with comparing confidence intervals of *d*s between the ideal-, self-, and actual partner ratings (Table S3). We found few cases of moderation by participant sex where ideal partner preferences correlated more (*z* = 3.59, *p* < .01) with actual partner 1 ratings in wave 2 in women (*r* = .57) than in men (*r* = .41). Women also had a larger discrepancy between ideal and self depressiveness in wave 1 (*d* = − 0.50 [95% CI: − 0.55, − 0.44]) than men (*d* = − 0.36 [95% CI: − 0.42, − 0.30]) and between self and actual partner 1 depressiveness in wave 1 (women: *d* = 0.17 [95% CI: 0.11, 0.23], men: *d* = − 0.03 [95% CI: − 0.10, 0.04]) and in wave 2 (women: *d* = 0.34 [95% CI: 0.26, 0.41], men: *d* = 0.12 [95% CI: 0.03, 0.21]).

**Table 2:** Comparison of the sexes’ ideal partner, self, and actual partner evaluations in depressiveness.

	Self	*n*	Mean	SD	t	Cohen’s d
Ideal partner wave 1	Women	1328	1.71	1.24	– 6.41***	– 0.25
	Men	1231	2.05	1.40		
Ideal partner wave 2	Women	988	1.86	1.27	– 4.59***	– 0.22
	Men	856	2.14	1.34		
Ideal partner wave 3	Women	796	1.87	1.23	– 3.92***	– 0.20
	Men	708	2.12	1.32		
Self-rating wave 1	Women	1328	2.50	1.58	– 2.52*	– 0.10
	Men	1231	2.66	1.62		
Self-rating wave 2	Women	1004	2.81	1.62	– 2.16*	– 0.10
	Men	863	2.98	1.63		
Self-rating wave 3	Women	812	2.73	1.58	– 1.34	– 0.07
	Men	722	2.84	1.57		
Actual partner 1 wave 1	Women	1109	2.18	1.49	– 4.63***	– 0.21
	Men	850	2.50	1.57		
Actual partner 1 wave 2	Women	725	2.16	1.42	– 4.34***	– 0.26
	Men	480	2.54	1.52		
Actual partner 1 wave 3	Women	543	2.21	1.46	– 2.10*	– 0.15
	Men	354	2.42	1.53		
Actual partner 2 wave 2	Women	139	2.25	1.57	– 1.96^†^	– 0.23
	Men	146	2.64	1.80		
Actual partner 2 wave 3	Women	68	2.51	1.69	– 1.14	– 0.19
	Men	84	2.85	1.84		

*SD*  Standard deviation

^†^*p* < 0.10, *p < 0.05, ***p < 0.001

Third, we compared the ideal and actual partner ratings of depressiveness in those who maintained a stable relationship throughout the study period, who broke up and remained single, who were initially single and found a relationship in wave 2, and those who broke up with their partner from wave 1 and started a new relationship in wave 2 (Table 3). To rely on an adequate number of participants for analysis, we focused on comparing the ratings in waves 1 and 2 and did not analyze wave 3. We found a discrepancy between ideal and actual partner 1 in those who stayed together in the same direction as in the overall sample, that is, the actual partner was more depressive than what they preferred (*d*s were − 0.33 in wave 1 and − 0.24 in wave 2). The difference in effect size was the largest among those who broke up after wave 1 (*d* = -0.62). Ideal and actual partners differed even in new relationships in wave 2 (*d* = -0.29), although, the difference was smaller and not statistically significant when compared to ideals stated in wave 1 (*d* = -0.07). Those who broke up with their partner 1 after wave 1 (and later found a new partner in wave 2) had a relatively large difference between their ideal at wave 1 and actual partner 1 (*d* = -0.44), and no difference between their ideal at wave 2 and actual partner 2 (*d* = -0.08). However, we found a difference when comparing the ideal at wave 1 and actual partner 2 (*d* = -0.30) in contrast to what we found in those who were initially single and then found a new relationship. After that, we compared the ideal partner preferences for depressiveness between waves 1 and 2 across the studied subgroups of different relationship maintenance to test whether the participants adjusted their ideals through time. We found differences only in the continually coupled participants, who adjusted their ideals between waves 1 and 2 by increasing their preferences for depressiveness closer to their actual partner (*n* = 1093, wave 1: *M* = 1.77, *SD* = 1.23, wave 2: *M* = 1.97, *SD* = 1.28, *d* = -0.14, *p* < .01).

**Table 3:** Ideal-actual partner differences in depressiveness across different relationship maintenance statuses.

Relationship maintenance		*n*	M (SD)	*r*	t	Cohen’s d
Stable relationship	Ideal partner wave 1	1098	1.76 (1.22)	0.49***	– 11.06***	– 0.33
	Actual partner 1 wave 1	1098	2.22 (1.49)			
	Ideal partner wave 2	1084	1.97 (1.28)	0.51***	– 7.98***	– 0.24
	Actual partner 1 wave 2	1084	2.30 (1.46)			
Breakup (then single)	Ideal partner wave 1	80	1.79 (1.06)	0.46***	– 5.55***	– 0.62
	Actual partner 1 wave 1	80	2.64 (1.49)			
New relationship (initially single)	Ideal partner wave 2	55	1.91 (1.25)	0.64***	– 2.18*	– 0.29
	Actual partner 2 wave 2	55	2.29 (1.66)			
	Ideal partner wave 1	55	2.16 (1.33)	0.27^†^	– 0.52	– 0.07
	Actual partner 2 wave 2	55	2.29 (1.66)			
Breakup (then new relationship)	Ideal partner wave 1	39	1.77 (1.40)	0.61***	– 2.73*	– 0.44
	Actual partner 1 wave 1	39	2.36 (1.61)			
	Ideal partner wave 2	36	2.11 (1.45)	0.51**	– 0.47	– 0.08
	Actual partner 2 wave 2	36	2.22 (1.44)			
	Ideal partner wave 1	36	1.72 (1.41)	0.33^†^	– 1.82^†^	– 0.30
	Actual partner 2 wave 2	36	2.22 (1.44)			

^†^*p* < .10, *p < 0.05, **p < 0.01, ***p < 0.001

### Classification of depressiveness trajectories

**Fig. 1:**
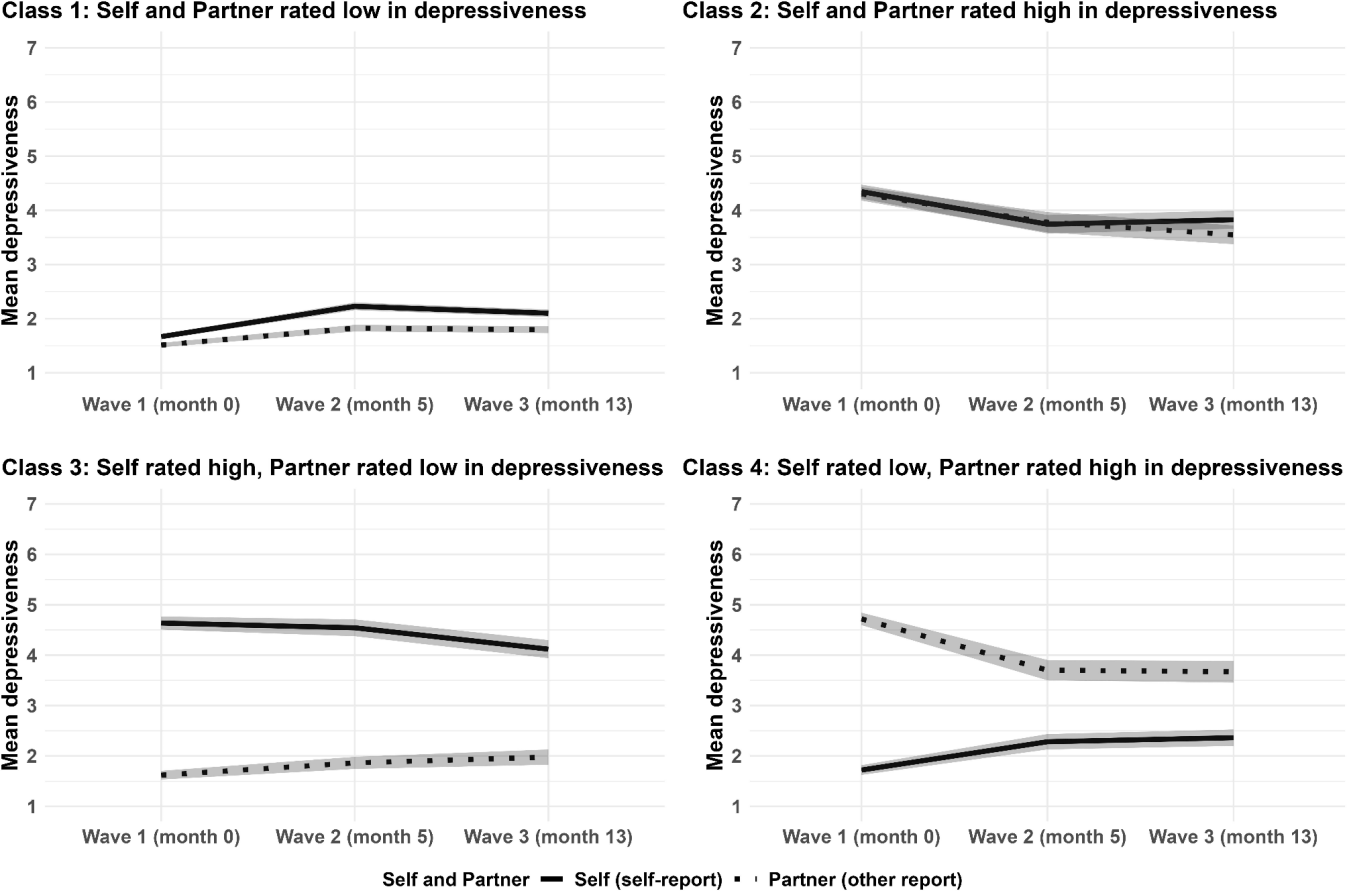
Four classes of couples based on depressiveness ratings of oneself and one’s actual partner (the grey shadowed areas represent 95% confidence intervals).

We conducted a “faux-dyadic” latent class growth modeling in Mplus (version 8.11) to identify distinct longitudinal trajectories in self and partner ratings of depressiveness. This is a probabilistic method that can detect patterns in the data and can categorize the participants into subgroups based on their most likely membership following similar trajectories. While we did not have actual dyads, the self and partner ratings were modelled as parallel processes as they would be in couples’ analyses. We exclusively relied on 1,943 participants who were coupled at any point in the study with one partner. See the details of the analysis in the Supplementary Methods. After evaluating a range of model indicators (Table S4), we selected the 4-class model for further investigation. The four identified classes were the following: Class (1) Self and partner rated low in depressiveness (59.44%), Class (2) Self and partner rated high in depressiveness (14.15%), Class (3) Self rated high, partner rated low in depressiveness (14.05%), and Class (4) Self rated low, partner rated high in depressiveness (12.35%; Fig. 1, Table S5). In Class 1, both self and partner had slightly increasing depressiveness (mean latent slope factor of self = 0.07, partner = 0.04). In Class 2, both self and partner had slightly decreasing depressiveness (self = − 0.07, partner = − 0.09). In Class 3, self had decreasing, partner had increasing depressiveness (self = − 0.05, partner = 0.05). In Class 4, self had increasing, and partner had decreasing depressiveness (self = 0.09, partner = − 0.14).

**Table 4:** Comparison of the four classes along demographic and relationship indicators.

	Class 1: Self and Partner rated low in depressiveness	Class 2: Self and Partner rated high in depressiveness	Class 3: Self rated high, Partner rated low in depressiveness	Class 4: Self rated low, Partner rated high in depressiveness	η^2^/V
Age, *M* (*SD*)	35.13 (9.10)	34.55 (9.53)	32.97 (8.85)	35.28 (9.33)	0.01**
Income (personal), *M* (*SD*)	4.06 (2.34)	3.77 (2.24)	3.45 (2.16)	4.06 (2.21)	0.01***
Income (household), *M* (*SD*)	5.11 (2.06)	4.65 (2.05)	4.92 (1.94)	4.96 (1.74)	0.01**
Men, *n* (%)	486 (42.08)	137 (49.82)	98 (35.90)	121 (50.42)	0.09**
New relationship, *n* (%)	37 (3.20)	10 (3.64)	6 (2.20)	2 (0.83)	0.05
Breakup + no new relationship, *n* (%)	41 (3.55)	17 (6.18)	8 (2.93)	14 (5.83)	0.06^†^
Breakup + new relationship, *n* (%)	25 (2.16)	4 (1.45)	5 (1.83)	5 (2.08)	0.02
Mate value, *M* (*SD*)	4.77 (1.22)	4.45 (1.39)	4.14 (1.46)	4.59 (1.33)	0.03***
Relationship seriousness, *M* (*SD*)	6.39 (1.21)	6.04 (1.40)	6.22 (1.41)	6.35 (1.10)	0.01**
Relationship satisfaction, *M* (*SD*)	6.00 (1.23)	5.13 (1.64)	5.57 (1.49)	5.31 (1.51)	0.06***
Partner’s mate value, (for self) *M* (*SD*)	6.17 (1.12)	5.52 (1.40)	5.87 (1.32)	5.55 (1.36)	0.05***
Partner’s mate value, (for others) *M* (*SD*)	5.46 (1.14)	4.94 (1.33)	5.14 (1.26)	5.04 (1.38)	0.03***
Ideal depressiveness at wave 1, *M* (*SD*)	1.41 (0.79)	3.26 (1.64)	1.70 (1.12)	2.21 (1.58)	0.25***

*SD*  standard deviation

^†^*p* < 0.10, **p < 0.01, ***p < 0.001

After that, we compared the four classes along selected relationship and demographic indicators (Table 4). We used one-way ANOVAs to compare continuous variables when homogeneity of variance was supported and Brown-Forsythe test when the variances differed (i.e., the self and partner’s mate value measures, relationship seriousness and satisfaction, and ideal preference for depressiveness). We used χ^2^ tests to compare dichotomous variables across the classes. We found the largest class differences in ideal partner preference for depressiveness at wave 1 (*η*^2^ = 0.25, high ideals in Classes 2 and 4 where the partner was depressive), relationship satisfaction (*η*^2^ = 0.06, high satisfaction in Class 1 where neither of them were depressive), participant’s rating of partner’s mate value (*η*^2^ = 0.05, low mate value of partner in Classes 2 and 4 where the partner was depressive, high in Class 1 where neither of them were depressive), self-perceived own mate value (*η*^2^ = 0.03, low mate value in Class 3 where the self was depressive, but the partner was not), and sex (Cramer’s *V* = 0.09, less men in Classes 1 and 3 where the partner was not depressive). We found weak evidence that Classes 2 and 4, where the partners were depressive, were more likely to break up without finding a new relationship (*V* = 0.06). We found no-to-negligible differences (*η*^2^ < 0.01) in sexual orientation, education, number of children, people in the household, size of residence, and number of relationships.

## Discussion

Using a large, longitudinal, and representative sample of Czech individuals, we tested two possible mechanisms responsible for couples’ similarity in depressiveness: preference for a self-similar partner and convergence over time. We first assumed that people may prefer and actively choose a self-similar partner, and perceived similarity between preferences and one’s actual partner will predict more stable relationships. Subsequently, we tested heterogeneity in the data looking for distinct patterns of self and partner depressiveness. We found that participants preferred someone less depressive than themselves, especially women. However, their actual partner was more depressive than their ideal partner with no sex differences. Concerning homogamy, we found a discrepancy between self-perceived depressiveness and perceived actual partner depressiveness that was especially pronounced in women evaluating their partner to be less depressive than themselves. The correlations were particularly strong between ideal and actual partner ratings and moderate between ideal and self, and self and actual partner ratings. Women preferred less depressive partners than men, and women perceived their partners to be less depressive than men rated themselves. Unexpectedly, women rated themselves slightly less depressive than men did, while men evaluated their partner’s depressiveness higher than women did. This contradicts robust evidence that found women more likely to be depressed and depressive than men^1,29^. However, this could be attributed to formulating the measure as “pessimism and depressiveness” instead of “depression” or simply “depressiveness”, as men and women are similar in pessimism^30,31^. Our findings also contradict the recent meta-analytical results that showed no sex bias in correlational accuracy of perception in romantic relationships and little mean-level bias in men but not in women^32^.

Altogether, we found perceived homogamy in the form of moderate correlations and small mean-level differences between self and actual partner ratings of depressiveness and little sex and no sexual orientation differences in these associations. Studies to date unequivocally agree that when one partner is diagnosed with depression (or is high in depressive symptoms, has been prescribed antidepressants, etc.), their partner has a higher probability of suffering from the same condition, and this has been demonstrated in various societies worldwide with varying effect sizes (*r*s = 0.15 to .19^8,17^; odds ratios [OR] = 2.91 to 5.54^7,9,33^; incidence rate ratio [IRR] = 1.58^6^).

The only study to date that tested active partner preference for negatively valenced personality traits reported no relationship between self-rating and preference for negative affectivity^13^. Importantly, even high-scorers in these traits rated them as rather undesirable in their potential partners, and their ratings were below the average of the scale. In contrast, we found moderate correlations between self-rated depressiveness and the preferred level of depressiveness in an ideal partner. However, our participants also gave a generally low average preference for pessimism and depressiveness (i.e., less than 2 on a 1 to 7 scale).

We also investigated whether the ideal-actual partner discrepancies differed depending on relationship maintenance. We found the largest discrepancy in those who broke up with their partners and then remained single and the second largest in those who broke up and found a new partner. In other words, individuals with a greater difference between their preferences and the perception of their actual mate choice were more likely to experience relationship dissolution. Those who were in a stable relationship adjusted their partner ideals between waves 1 and 2 to be closer the perception of their actual partner, which underscores the presence and even functionality of positivity biases in romantic relationships^34^. Curiously, those who were initially single found a partner in wave 2 just as their ideals were in wave 1 but not their ideals in wave 2. In contrast, in cases where individuals broke up after wave 1 and then found a new partner in wave 2, the perception of their actual partners matched their ideal preferences in wave 2 but not in wave 1. Perhaps the relationship dissolution influenced changes in partner preferences for depressiveness differently than finding a partner after being single for a while, but replication and more evidence are needed to be conclusive.

Further, we identified four distinct classes of longitudinal depressiveness trajectories across 13 months. We found the largest class (59%) to be both self and actual partner perceived to be low in depressiveness. They reported the highest relationship satisfaction, self-rated and partner’s mate value, and the lowest preference for depressiveness. In the second class (14%) both self and actual partner had high perceived depressiveness. They were the most likely to break up and remain single between the first two waves. They also had the lowest relationship satisfaction and partner’s mate value among the four classes. Their preference for depressiveness was the highest, however. This may explain why they were together (i.e., they preferred a depressive partner), or they may have adjusted their preferences closer to the perception of their actual partner^34^. The third class (14%) had high perceived depressiveness in self and low in partner. They were the least likely men among all four groups and the least likely breaking up with no new relationship. Self-rated mate value was the lowest in this class with a medium relationship satisfaction and partner’s mate value. The last, fourth class (12%) had low perceived depressiveness themselves and high perceived depressiveness in their partner. Their relationship satisfaction was relatively low, together with the partner’s mate value, a likely reason why these relationships more frequently dissolved (not finding a relationship subsequently) than Classes 1 and 3. Importantly, class differences in being in a new relationship were inconclusive, but Class 4 couples were the least likely partners of a newly formed relationship.

Our results are in line with studies that classified couples based on their longitudinal depression as dyads. Volling et al. classified about one year long peripartum depression trajectories of 231 families welcoming their second child^35^. They found similar trajectories as we did: both high, both low, mother high father low, father high mother low. Both depressed parents had more marital negativity and parenting stress than both non-depressed parents, while families with one depressed parent had moderate levels of marital negativity. Another study was conducted on over eleven thousand European older couples’ depressive symptoms that were recorded every 2 years for 12 years^36^. The constantly non-depressed Class 1 here was found there too; and the mostly female depressed self, non-depressed partner Class 3 coincides with a class of couples identified there. Previous results thus confirm a heterogamous depression pattern, where couples’ dissimilarity persists over time, and yet their relationship is stable. In contrast to theories that would predict homogamy to be advantageous for heightened relationship satisfaction in a relationship^37^, we see that the two stable relationship classes are when both are non-depressed (homogamy), or only the woman is depressed (heterogamy), and the unstable relationship is when both are depressed (homogamy). This of course assumes that evidence related to “depressiveness” can be generalized to inferences on “depression”^15,38^.

In the homogamous classes (i.e., both depressive, both non-depressive), the couples changed in the same direction (i.e., both decreased or both increased), albeit with a different rate of change. In the heterogamous classes (i.e., couples with low perceived similarity), we observed that partners tend to slowly converge, possibly from a contagion effect^39^. That is, those couples who were already perceived to be similar (i.e., both had low or high depressiveness) did not converge, rather diverged a little, which is consistent with previous research^36^. In contrast, in relationships where self and partner were perceived to be especially different from each other (i.e., one of them had low, the other had high depressiveness), they changed towards each other. With their rate of change, it would take more than 6 years for self and partner in Class 4 to meet at the same level of depressiveness. In contrast, in Class 3, if the rate of change remains so slow, self and partner would not become similar in depressiveness until at least 14.5 years (assuming the change is linear). They may even stay together for this long, as Class 3 had one of the lowest dissolution rates. So maybe reaching perceived similarity over a longer period is more beneficial for the relationship than other routes of perceived similarity in depressiveness (e.g., perceiving to be similar in high depressiveness from the beginning, or both becoming depressive suddenly because of a shared stressful life event).

We did not find a systematic convergence overall. However, we identified two sub-groups in our sample that did converge, albeit slowly. There is limited evidence for convergence in traits generally^12^, but previous research is mixed when studying convergence in depression, especially because of differences in the study methods. For example, no correlation was found between relationship duration and dyad concordance in depressive symptoms^17^. Similarly, no crossover effects were reported in older couples^16^. That is, the focal individual’s depression was not associated with the growth of depressive symptoms in their partner in the following years. Conversely, emotional contagion was observed in depression^39^, even though research is equivocal about the magnitude of the sexes’ relative influence on each other^18,21,22^. Also, individual differences moderate these findings, as people with high empathic accuracy resemble their partners more in depressive symptoms^40^. Further, not all couples are the same, in about 7% of couples both men and women had convergent decreasing depressive symptoms, but also in about 8%, both had divergent increasing depressive symptoms^36^. In summary, in relatively malleable characteristics such as depression, partners can influence each other considerably. Conversely, while we studied depressiveness instead of depression, a supposedly more stable form of negative affectivity, we still found hints of convergence. These patterns should be taken into consideration in mental health services. For example, interventions targeting improved empathic accuracy could lead to enhanced contagion in depression, which should be avoided generally as a possible iatrogenic harm^40^.

In essence, we found supporting evidence for both preference for self-similarity and convergence in depressiveness, but with caveats. For instance, the preference for depressiveness can be a consequence of preference for another trait that correlates with depressiveness (i.e., secondary assortment^17^). Alternatively, geographical and social proximity can also cause passive positive assortment. When partners meet based on physical proximity (e.g., attending the same university, the same religious masses), if these backgrounds correlate with the trait under study (e.g., intelligence, attitudes), then concordance in these traits is a passive result of these forces^12^. Importantly, even though we showed correlations between self- and ideal preference ratings, we cannot rule out the possibility that, for example, preference for optimism, warmth, or general health drive these correlations as underlying unaccounted factors. Similarly, we do not have information about the participants’ hobbies or preferences for spending their free time, which could result in perceived similarity due to passive assortment. Clearly, the individual homogamy mechanisms are not mutually exclusive^41^.

Additionally, mating market operations are also a likely influence^12^. In this homogamy mechanism, couples form relationships if they are similar in their mate value (i.e., how much they find each other desirable). However, when two individuals are not similarly desirable, the risk of jealousy is arising in the less desirable partner and, hence, the chance of relationship dissolution increases. Mating market operations thus cause similarity in mate value and relatedly in many characteristics because of mutual attraction being necessary for forming long-term relationships. Because we know that mate value is negatively correlated with depression^42,43^, homogamy in depressiveness may be a result of the combination of active and passive secondary assortment and market operations too.

We found that a likely consequence of homogamy (or the lack of it) in depressiveness is differential relationship maintenance. Further, concerning genetics, couples’ similarity is beneficial for genes as this way their offspring will be more like them than 50%^44^. Indeed, Cabrera-Mendoza et al. found that partners tend to resemble each other in possessing genetic alleles predisposing to various mental disorders (i.e., polygenic risk score correlation), including depression, albeit with very small effect sizes (*r*s = 0.01 to 0.03)^45^. The social consequences of homogamy in depressiveness are likely enhancing difficulties in social mobility, especially if both are depressed and experience associated economic hardship^46,47^. On the other hand, they may experience increased intimacy if they are depressed^2^ and maybe higher relationship satisfaction too through similarity^37^ (but see^48^).

### Limitations and future directions

We relied on a representative, longitudinal, socioeconomically diverse sample from an underrepresented Central European country. However, to conform to restrictions on the number of items we could measure, we used an overly simple single-item measure of “pessimism and depressiveness”. Even so, researchers often successfully rely on single-item measures for their face-validity and brevity^49^. Additionally, it remains unclear how well our findings generalize from depressiveness to depression and vice versa. While depression contagion is well-documented^39^, personality trait convergence is less likely to occur over a short period. This may have resulted in weaker convergence effects than what we would expect if measuring depression rather than depressiveness. Also, data were collected from individuals who rated their own perceptions of themselves and their actual/ideal partners (i.e., perceived homogamy), raising questions about rating accuracy and potential projection. Incorporating data from both partners (i.e., actual homogamy) would provide deeper insights into these dynamics from a dyadic perspective.

While our study provided sufficient variance in the age-range of 18 to 50, a developmental perspective suggests that examining mating behavior in teenagers and older adults could provide additional insights. Although the longitudinal design with multiple assessment points is a strength of the study, it is possible that longer follow-up (e.g., 5 years, 10 years) would produce different results. Longitudinal data capturing actual mate choice and relationship dissolution is valuable and scarce. Still, only a few participants were single, found a new relationship, or broke up with their partners during a mere year of follow-up. On the other hand, the relatively low frequency of singlehood is because of the representativeness of our data; that is, the population in general is more likely coupled than not. Further, we did not have data consistently available on the coupled participants’ relationship types (e.g., dating, married). Future research should investigate how different commitment levels in the relationship influence the tested associations. Last, it was generally difficult to reliably track their relationship maintenance because of incomplete follow up data and because of the naivety of the participants. Nonetheless, our study is an important first step in studying the influence of mate choice on couples’ perceived similarity in depressive traits, and our evidence supports the need for future research relying on better measures and more single participants actively looking for a partner.

## Conclusions

In sum, preference for similarity and convergence in depressiveness may be responsible for the widely observed couples’ similarity in depression, but likely not the only influencing factor. Those who broke up had the largest ideal-actual partner discrepancy in depressiveness; and those who stayed together adjusted their preferences closer to the perception of their actual partner. The most stable relationships were both non-depressive, or only the self was depressive and the partner non-depressive. Thus, homogamy and heterogamy in depressiveness complexly influenced relationship satisfaction and stability. Future research should explore why heterogamy may lead to greater relationship stability compared to consistently high depressiveness homogamy.

## Methods

### Procedure and participants

We conducted a nationally representative three-wave online study in Czechia. The data was collected by the Czech company, National Panel. The participants gave their informed consent via tick-box. All participants were paid for their participation in each wave (≈ 5 EUR worth in CZK). The project was approved by the ethical committee of the Second Faculty of Medicine, Charles University, Prague (Approval No. EK-291.1.8/21) and adhered to the Declaration of Helsinki.

The total sample included 2,793 participants (50.4% women, ) aged at baseline between 18 and 50 years (*M*_age_ = 34.21, *SD* = 9.43). Participants were recruited based on quotas for sex, age, education, residential size, and region (refer to Table S1 for details). Adhering to company policy, we did not ask participants their sexual orientation, so we gathered data from their relationship experiences and preferences (i.e., past and current relationships with and preferences for same- and/or opposite-sex individuals). Most participants lived in heterosexual relationships (94.3%), 5.7% had non-heterosexual interests. Their education was rated from 1 (*elementary or unfinished*) to 5 (*university education; M* = 3.11, *SD* = 1.24), their personal income from 1 (*less than 10*,*000 CZK*) to 10 (*60*,*001 CZK and more*,* M* = 3.82, *SD* = 2.29), their household income from 1 (*less than 10*,*000 CZK*) to 11 (*100*,*001 CZK and more*,* M* = 4.87, *SD* = 2.13). They had between zero and five children (*M* = 0.92, *SD* = 1.06), with between one and nine people living in their households (*M* = 2.98, *SD* = 1.26). On average, participants had in their lifetime 7.36 romantic relationships (*SD* = 12.20) and 8.97 sex partners (*SD* = 15.05).

In the first wave in June 2021, 2,559 people completed the questionnaire, in the second wave in November 2021, 1,951 people took part (out of which 234 people were new recruits to the study). In the third wave in July 2022, 1,591 people participated. In all three waves 1,200 people took part, 908 in two waves (not necessarily consecutive waves), and 685 in one wave.

Most participants (69.6%, *n* = 1,943) had a partner during the study period at any point; their baseline length of relationship was, on average, 9.96 years long (*SD* = 8.88). Throughout the study 20.3% were single (*n* = 568, no record of any relationship) – this pertains to participants who took part in any number of the three waves. Only 55.2% (*n* = 1,543) participants had reliably interpretable relationship status *maintenance*. The rest of the participants were not possible to categorize as a result of incomplete follow-up, or in some cases we had to assume the participants misunderstood the questions about still being together with their partner from the previous waves. Demonstrably 39.5% (*n* = 1,103) had a stable relationship (maintained for at least two waves). Continually single were 9.5% (*n* = 266, singlehood maintained and documented for at least two waves); 2.0% (*n* = 55) were initially single but started a new relationship between waves 1 and 2. Few people experienced relationship dissolution between waves 1 and 2, 1.4% (*n* = 39) initiated a new relationship afterwards and 2.9% (*n* = 80) did not initiate a new relationship subsequently. We focused on new and dissolved relationships between waves 1 and 2 to maximize the participants for comparisons (i.e., too few people changed relationship status between waves 2 and 3).

### Measures

Participants rated “How desirable/attractive do you think you are to others as a potential partner?” (i.e., self-perceived own mate value; 1 = *very undesirable*, 7 = *very desirable; M* = 4.50, *SD* = 1.34). We intended to measure desirability; however, the Czech expression we used (přitažlivý) means something between “attractive” and “desirable”. Participants also rated “How serious is your relationship?” (i.e., relationship seriousness; 1 = *non-committed*, 7 = *very serious; M* = 6.39, *SD* = 1.28); “How satisfied are you in your relationship?” (i.e., relationship satisfaction; 1 = *very unsatisfied*, 7 = *very satisfied; M* = 5.69, *SD* = 1.44); “How desirable/attractive is your partner to you?” (i.e., partner’s mate value for self; 1 = *very undesirable*, 7 = *very desirable; M* = 5.94, *SD* = 1.27); “How desirable/attractive do you think your partner is to others?” (i.e., partner’s mate value for others; 1 = *very undesirable*, 7 = *very desirable; M* = 5.29, *SD* = 1.24).

The participants also rated “How well do the following characteristics describe your ideal partner/actual partner/you?”. Here, among other characteristics, the participants rated how “pessimistic and depressive” (in Czech: pesimistický a depresivní) their ideal partners, their actual partners, and themselves were in each wave (1 = *not at all*, 7 = *very much*). Those who were initially coupled but broke up rated their former partner (not analyzed in this study) in the subsequent wave together with their new partner if they had one. Initially single participants who found someone later rated their new partners, and the new recruits also rated their partners if they had one in wave 2. Importantly, the partners from wave 1 were labelled as actual partner 1, the partners “joined” in wave 2 were labelled as actual partner 2 (either because they were a new partner, even a secondary new partner, or because they were the partners of new recruits). The participants also completed additional questionnaires not used in this analysis, as the study was part of a larger project.

## Electronic supplementary material

Below is the link to the electronic supplementary material.

Supplementary Material 1

## Data Availability

Data and syntax for the present analysis are available on the Open Science Framework: https://osf.io/pvd29/?view_only=d14e75c99b78483792002d5810e00647. This study was not preregistered.
