# Supplementary material for: Mechanisms creating homogamy in depressiveness in couples: A longitudinal study from Czechia
Source: Sci Rep. 2025 Mar 17;15:9143. doi: 10.1038/s41598-025-93065-7 (PMC11914466; doi:10.1038/s41598-025-93065-7)
Supplement: Supplementary file 1 — Supplementary Material 1 [file 41598_2025_93065_MOESM1_ESM.pdf]

Mechanisms creating homogamy in depressiveness in couples: A longitudinal study from Czechia

Zsófia Csajbók*, Jakub Fořt, Peter K. Jonason, Jan Havlíček, Jakub Binter, & Zuzana Štěrbová

*Corresponding author: Zsófia Csajbók, Ph.D., Faculty of Humanities, Charles University, Pátkova 5, Prague 8, 182 00, Czech Republic. **Email:** zsofia.csajbok@fhs.cuni.cz

Supplementary Methods

First, we fitted a latent growth model against the self and partner ratings across the three waves with 5 and 8 months lags specified between the three waves. The model with correlated latent slope and intercept factors between the parallel processes of the self and partner ratings, fixed variance of the latent slope factor of the partner, and correlated self-partner observations at each wave fitted excellently ($\chi^2(8) = 32.34$, RMSEA = .04 (90% CI .03-.05), CFI = .99, TLI = .98, SRMR = .03, Brown, 2006). After that, we extracted 2, 3, 4, 5, and 6 latent classes. We submitted the fitted parallel processes model into latent class growth modeling (LCGM), where the variances of the latent growth factors were fixed at zero. We chose LCGM over growth mixture modeling (GMM) where the latent growth factors are held equal across the classes, because the estimation of the classes with GMM produced estimation errors. We relied on selected model indicators when deciding on which solution to choose. The selected indicators were Akaike Information Criterion (AIC; Akaike, 1973), Bayesian Information Criterion (BIC; Schwartz, 1978), entropy, the Vuong-Lo-Mendell-Rubin Likelihood Ratio Test (VLMR LRT; Vuong, 1989), the Lo-Mendell-Rubin Adjusted Likelihood ratio test (LMR ALRT; Lo et al, 2001), and the parametric bootstrapped likelihood ratio test (BLRT; McLachlan & Peel, 2004). These indicate whether extracting k+1 classes (starting with k = 1, that is, the sample is homogenous) more meaningfully describes the data than extracting k classes. The best solution should have the lowest AIC and BIC, entropy over .70, and significant VLMRT LRT, LMR ALRT, and BLRT tests (Van De Schoot et al., 2017). All solutions were replicated with different number of random starts. The best loglikelihood values were always replicated multiple times.

Considering these test results and the interpretability/parsimony of the solutions, we selected the 4-class solution to report (Supplementary Tables S4 and S5). Although the 5- and 6-class solutions were technically supported by the model indicators, the interpretation of the solutions did not provide a more meaningful classification of the participants in the 5- and 6-class

solutions than in the 4-class solution. That is, the 4 classes were sufficiently different, while the 5th and 6th classes did not have clearly distinctive and recognizable features considering that the classification was done on a single-item measured from 1 to 7. The entropy was also slightly lower in the 5-class than in the 4-class solution, further, the class sizes also started to become very small by extracting more classes (small subgroups of 6%, about $n = 100$), which may not be a trustworthy representation of the given patterns. For these reasons, we remained with the 4-class, more certain distinction between the classes, although a replication would be helpful in deciding which solution is more meaningful and reliable. See all the 2- to 6-class solutions in Supplementary Figure S1a-e.

Supplementary Table S1. Frequency of participants living in different sizes of residence and having different levels of education

Size of residence		Frequency	Percent	Valid Percent
Valid	≤ 1,000 residents	405	14.5	15.8
	1,001-5,000 residents	480	17.2	18.8
	5,001-20,000 residents	466	16.7	18.2
	20,001-50 000 residents	322	11.5	12.6
	50,001-100,000 residents	260	9.3	10.2
	100,000 residents	626	22.4	24.5
	Total	2559	91.6	100.0
Missing		234	8.4	
Total		2793	100.0	

Highest level of education		Frequency	Percent	Valid Percent
Valid	Elementary (even unfinished)	205	7.3	7.3
	High school without a diploma	725	26.0	33.3
	High school with diploma	1105	39.6	72.9
	Higher professional	80	2.9	75.7
	University	678	24.3	100.0
	Total	2793	100.0	

Supplementary Table S2. Correlations among ideal partner, self, and actual partner ratings in the first wave across non-heterosexuals and heterosexuals (Fisher's *z* tests indicated no group differences)

Non-heterosexuals		Self-rating	Actual partner
Ideal partner	*r*	.303***	.545***
	n	139	92
Self-rating	*r*	–	.251*
	n		92
**Heterosexuals**		**Self-rating**	**Actual partner**
Ideal partner	*r*	.395***	.498***
	n	2420	1867
Self-rating	*r*	–	.298***
	n		1867

* $p < .05$. *** $p < .001$.

Supplementary Table S3. Comparison of women's and men's correlation coefficients and Cohen's *d* coefficients

			Women				Men				Sex comparison	
			n	*M* (*SD*)	*r*	Cohen's *d* (95% CI)	*n*	*M* (*SD*)	*r*	Cohen's *d* (95% CI)	*r* diff. (*z*)	*d* larger in women?
Ideal vs Self	Ideal	Wave 1	1328	1.71 (1.24)	.39***	-0.50*** (-0.55, -0.44)	1231	2.05 (1.40)	.38***	-0.36*** (-0.42, -0.30)	0.30	yes
	Self		1328	2.50 (1.58)			1231	2.66 (1.62)				
	Ideal	Wave 2	988	1.86 (1.27)	.30***	-0.55*** (-0.61, -0.48)	856	2.14 (1.34)	.34***	-0.49*** (-0.56, -0.42)	-0.95	no
	Self		988	2.81 (1.62)			856	2.97 (1.63)				
	Ideal	Wave 3	796	1.87 (1.23)	.35***	-0.53*** (-0.60, -0.45)	708	2.12 (1.32)	.39***	-0.44*** (-0.52, -0.37)	-0.90	no
	Self		796	2.71 (1.56)			708	2.83 (1.57)				
Self vs Partner	Self	Wave 1	1109	2.48 (1.57)	.30***	0.17*** (0.11, 0.23)	850	2.45 (1.51)	.30***	-0.03 (-0.10, 0.04)	< .01	yes
	Partner 1		1109	2.18 (1.49)			850	2.50 (1.57)				
	Self	Wave 2	725	2.79 (1.59)	.24***	0.34*** (0.26, 0.41)	480	2.75 (1.51)	.30***	0.12* (0.03, 0.21)	-1.10	yes
	Partner 1		725	2.16 (1.42)			480	2.54 (1.52)				
	Self	Wave 3	543	2.69 (1.56)	.33***	0.28*** 0.19, 0.36)	354	2.57 (1.48)	.34***	0.08 (-0.02, 0.19)	-0.16	no
	Partner 1		543	2.21 (1.46)			354	2.42 (1.53)				
	Self	Wave 2	139	2.79 (1.66)	.35***	0.29** (0.12, 0.46)	146	2.84 (1.58)	.32***	0.10 (-0.07, 0.26)	0.28	no
	Partner 2		139	2.25 (1.57)			146	2.64 (1.80)				
	Self	Wave 3	68	2.65 (1.60)	.62***	0.09 (-0.15, 0.33)	84	2.70 (1.50)	.49***	-0.08 (-0.30, 0.13)	1.13	no
	Partner 2		68	2.51 (1.69)			84	2.85 (1.84)				
Ideal vs Partner	Ideal	Wave 1	1109	1.68 (1.22)	.49***	-0.36*** (-0.42, -0.30)	850	1.97 (1.37)	.49***	-0.36*** (-0.43, -0.29)	< .01	no
	Partner 1		1109	2.18 (1.49)			850	2.50 (1.57)				
	Ideal	Wave 2	723	1.86 (1.26)	.57***	-0.23*** (-0.31, -0.16)	479	2.15 (1.35)	.41***	-0.25*** (-0.34, -0.16)	3.59***	no
	Partner 1		723	2.15 (1.41)			479	2.53 (1.52)				
	Ideal	Wave 3	543	1.88 (1.23)	.49***	-0.24*** (-0.33, -0.16)	353	2.05 (1.30)	.56***	-0.28*** (-0.39, -0.17)	-1.41+	no
	Partner 1		543	2.21 (1.46)			353	2.42 (1.53)				
	Ideal	Wave 2	139	2.02 (1.48)	.59***	-0.17+ (-0.33, < 0.01)	146	2.25 (1.59)	.50***	-0.23** (-0.39, -0.07)	1.07	no
	Partner 2		139	2.25 (1.57)			146	2.64 (1.80)				
	Ideal	Wave 3	68	2.09 (1.50)	.64***	-0.31* (-0.56, -0.07)	84	2.26 (1.48)	.55***	-0.36** (-0.58, -0.14)	0.84	no
	Partner 2		68	2.51 (1.69)			84	2.85 (1.84)				

Note. *r* diff. = Fisher's *r* comparison test.
+ p < .10. * p < .05. ** p < .01. *** p < .001.

Supplementary Table S4. Model parameters and results of the 2-, 3-, 4-, 5-, and 6-class latent class growth models

N of classes	Best Loglikelihood	AIC	BIC	Entropy	VLM RLR	LMR ALRT	BLRT
2	-14220.864	28477.729	28578.024	.858	$p < .001$	$p < .001$	$p < .001$
3	-13901.483	27848.965	27977.121	.812	$p < .001$	$p < .001$	$p < .001$
4	-13685.639	27427.279	27583.294	.864	$p < .001$	$p < .001$	$p < .001$
5	-13583.579	27233.158	27417.033	.860	$p = .001$	$p = .001$	$p < .001$
6	-13488.034	27052.068	27263.804	.876	$p = .026$	$p = .029$	$p < .001$[a]

Note. AIC=Akaike information criterion; BIC=Bayesian information criterion; VLM RLT=Vuong-Lo-Mendell-Rubin likelihood ratio test; LMR ALRT=Luo-Mendell-Rubin adjusted likelihood ratio test; BLRT=bootstrap likelihood ratio test.

[a] The best loglikelihood value was not replicated in the LRT starts option even after increasing the random starts to 2000 500.

Supplementary Table S5. Class proportions and mean intercept and slope results in the 4-class dyadic latent class growth model

	N of class members	% of total *N*	Mean latent intercept factor of self	Mean latent slope factor of self	Mean latent intercept factor of partner	Mean latent slope factor of partner
Class 1	1155	59.44%	1.705***	0.068***	1.524***	0.044***
Class 2	275	14.15%	4.270***	-0.074**	4.231***	-0.089***
Class 3	273	14.05%	4.588***	-0.050*	1.632***	0.046***
Class 4	240	12.35%	1.775***	0.090**	4.624***	-0.136*

* $p < .05$. ** $p < .01$. *** $p < .001$

a

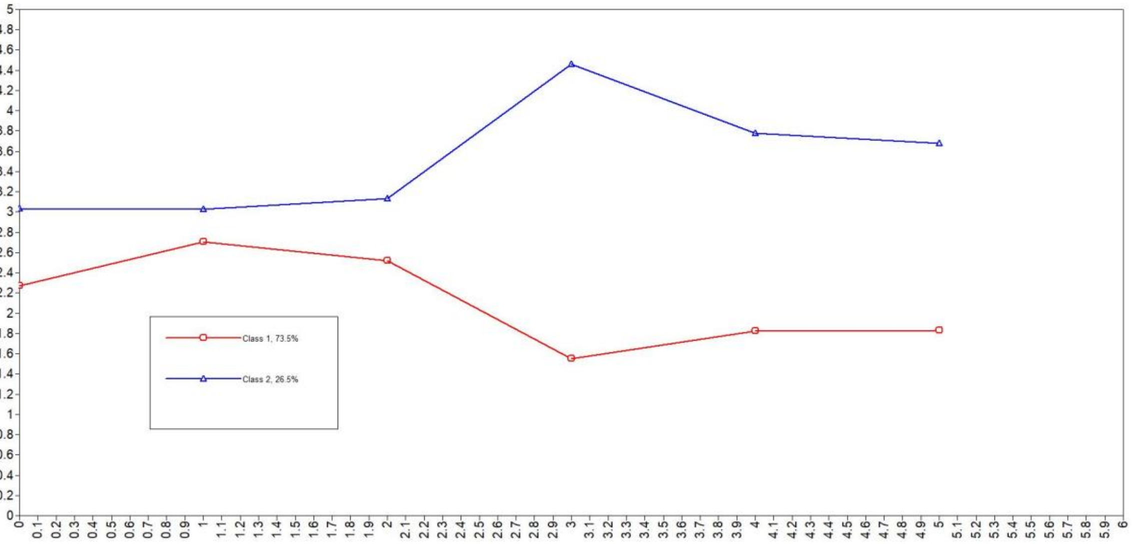

b

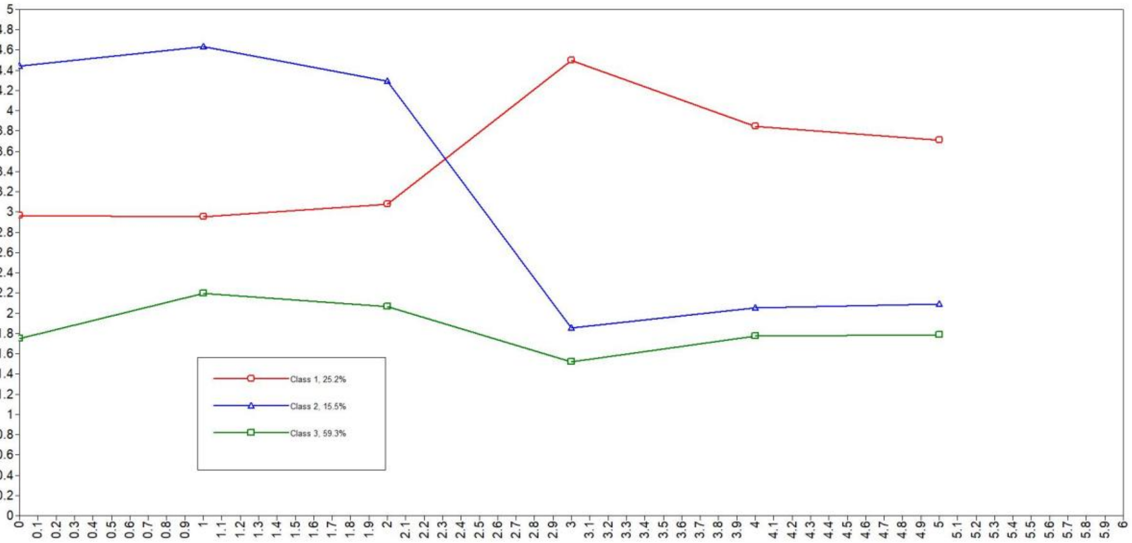

c

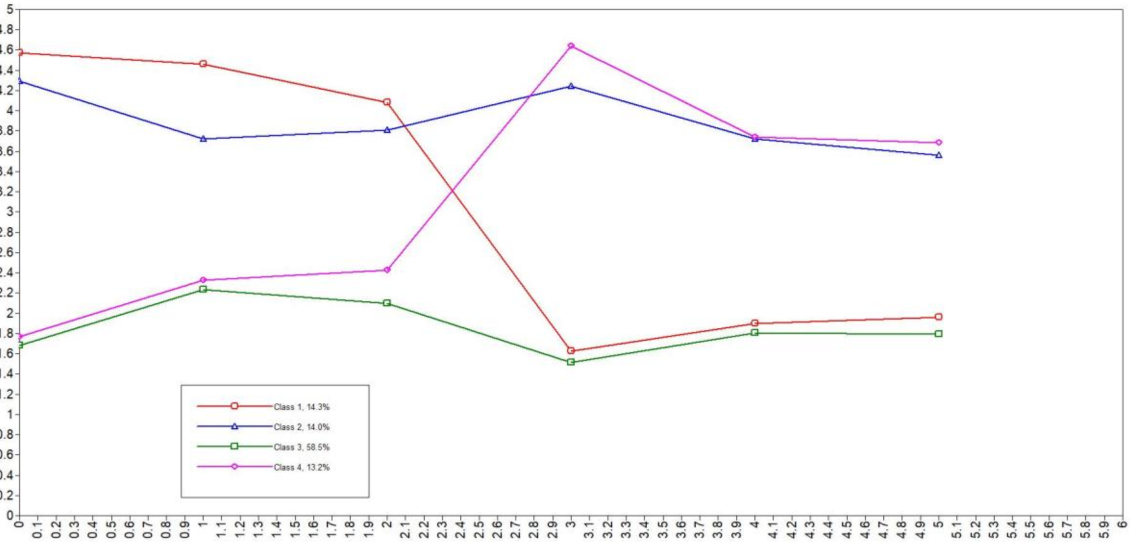
Class 1, 14.3%
Class 2, 14.0%
Class 3, 58.5%
Class 4, 13.2%

d

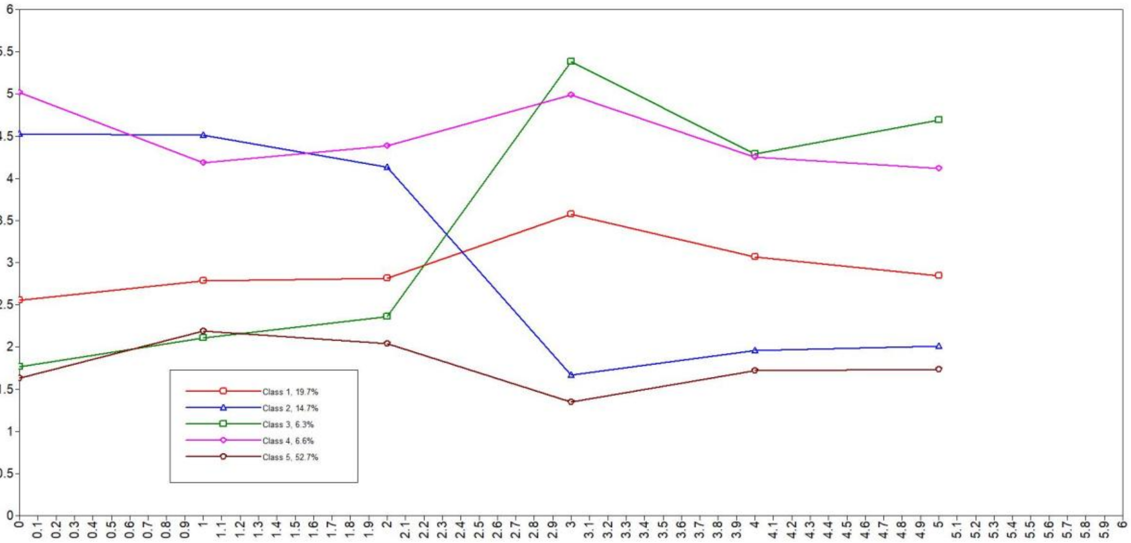
Class 1, 19.7%
Class 2, 14.7%
Class 3, 6.3%
Class 4, 6.6%
Class 5, 52.7%

e

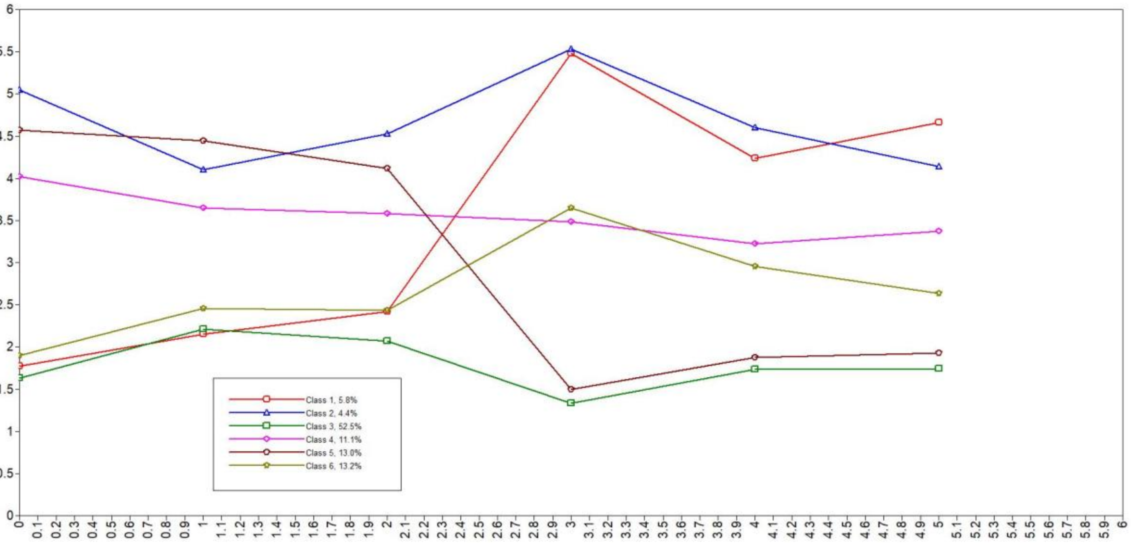

Supplementary Figure S1. Mean depressiveness of self and partner (left: 0, 1, and 2 points on the x axis; and right: 3, 4, and 5 points on the x axis within each diagram, respectively) in each model extracting 2 to 6 classes. **a**: 2-class model; **b**: 3-class model; **c**: 4-class model; **d**: 5-class model; **e**: 6-class model.

References

Akaike, H. (1973). Information theory as an extension of the maximum likelihood principle. In B. N. Petrov & F. Csaki (Eds.), *Second international symposium on information theory* (p. 267). Budapest, Hungary: Akademiai Kiado.

Brown, T. A. (2006). *Confirmatory factory analysis for applied research.* Guilford Press.

Lo, Y., Mendell, N., & Rubin, D. (2001). Testing the number of components in a normal mixture. *Biometrika, 88*, 767–778.

McLachlan, G., & Peel, D. (2004). *Finite mixture models*. New York, NY: Wiley.

Schwarz, G. (1978). Estimating the dimension of a model. *The Annals of Statistics, 6,* 461–464.

Van De Schoot, R., Sijbrandij, M., Winter, S. D., Depaoli, S., & Vermunt, J. K. (2017). The GRoLTS-checklist: guidelines for reporting on latent trajectory studies. *Structural Equation Modeling: A Multidisciplinary Journal*, *24*, 451-467.

Vuong, Q. (1989). Likelihood ratio tests for model selection and non-nested hypotheses. *Econometrica, 57*, 307-333.
